# Embalmment by Injection

**Published:** 1845-11

**Authors:** 


*Embalmment by Injection.*—Dr. Broc, aided by Dr. Pouzin, embalmed Marshal d'Erlon, by means of injection through the carotid artery. Five hundred grammes of corrosive sublimate were dissolved in two thousand grammes of alcohol (rather more than a quart). Twenty-five grammes of arsenious acid were dissolved in a quarter of a pint of hot water. Four grammes of the essence of cloves, fifteen grammes of the essence of lavender, and five grammes of the essence of neroli (orange-flowers), were dissolved in a quart of alcohol. The three solutions were mixed together at the time the injection was made. Three-fourths of the mixture were thrown in at the lower end of the left common carotid artery. The remainder was injected into the cavities of the pleuræ and peritoneum. The gas contained in the intestines was removed by means of punctures. (The body was afterwards wrapped up in linen bandages). It is important not to throw more than three pints of a liquid into the body of an adult, as otherwise the injection will exhale by the bronchia, and return freely through the mouth.

The following is the liquid which appears to me to be best adapted to preserve bodies:—Four pints of alcohol, one hundred grammes of creosote, one hundred grammes of the binioduret of mercury, and two hundred grammes of the ioduret of of potassium.—*Ibid.*

